# Supplementary material for: Identification of Emerging Hazards in Mussels by the Galician Emerging Food Safety Risks Network (RISEGAL). A First Approach
Source: Foods. 2020 Nov 10;9(11):1641. doi: 10.3390/foods9111641 (PMC7697966; doi:10.3390/foods9111641)
Supplement: Supplementary file 1 [file foods-09-01641-s001.zip › Tables_figures_supplementary/Table S1_supplementary.docx]

| Table 1. *Groups of concepts* used in non-scientific survey. |
| --- |
| **Group 1 (Agents):** vir*, hepatitis E,Aichi virus*, norovirus*, sapovirus*, parasit*, giardia*, toxoplasma*, cryptosporidi*, angiostrongyl*, anisak*,poison*, Toxin*, algal toxin*, red tide*, tetrodotoxin*, ciguatoxin*, domoi*, okadai*, cyclic imin*, brevetoxin*, palitoxin*, azaspiracid*, contaminan*, pollutan*, chemical*, pharmaceutic*, element*, pollutan*, element*, endocrine disrup*, polycycli*, PAH*, retardan*, halogenate*, polychlorinat*, PCB*, pesticide*, microplast*, bacteri*, vibrio*, vibrio parahaemol*, vibrio cholera*, arcobacte*, antimicrobial resist*, antibiotic resist*, resistance to anti*, listeri*, clostridi*, salmonel*, escherich*, lactococc*. |
| **Group 2 (General words):** hazard*, danger*, peril*, risk*, threat*, problem*, pathogen*, zoono*, intoxicat*, infect*, migr*, safe*, issue*, health*, disease*, ill*, sick*, outbreak*, mortal*, death*, morbid*, inciden*, prevalen*, gastroenter*, colit*, diarrhe*, choler*, alert*, epidem*, pandem*, global*, global market*, climate chang*, clima*, chang*, new food*, migr*, age*. |
| **Group 3 (Food products)**: seafood*, shellfish*, mollus*, bivalve*, clam*, oyster*, mussel*, cockle*, scallop* |
