# Supplementary material for: Identification of Emerging Hazards in Mussels by the Galician Emerging Food Safety Risks Network (RISEGAL). A First Approach
Source: Foods. 2020 Nov 10;9(11):1641. doi: 10.3390/foods9111641 (PMC7697966; doi:10.3390/foods9111641)
Supplement: Supplementary file 1 [file foods-09-01641-s001.zip › Tables_figures_supplementary/Table S10_supplementary.docx]

**TABLE 10: FORM USED BY THE PRIORITIZATION OF EMERGING HAZARDS**

**Researcher: Date:**

1. **Proposed emerging issue** (Short description)
2. **Additional information**
   1. Information source (choose one): Scientific journal, Scientific outreach journal, Newspaper/Magazine, Official webs, Other Webs/Blogs, Digital platforms
   2. Restrictions associated with the issue evaluation (write them)
   3. Effects on health (indicate)
   4. Relevant comments
   5. Indicate if the issue is described in another food/habitat/country
3. **Assessment**
   1. Agent
   2. Is it a new hazard? Yes/No
   3. Classify the issue (choose one): New hazard, Increased exposure, Increased susceptibility, New scenario
   4. Driving factors (choose one or write it): environmental factors, human activities, human consumption habits, migration.
   5. Risk groups (choose one or write it): any, middle age population, ederly, youth people, inmunosupressed, allergin, in between others.
   6. Imminence (choose one): short (1-3 years), medium (3-10 years) or large (>10 years).
   7. How probable you consider the appearance of the issue in Galicia? (choose one): highly likely, slightly likely, no enough data available.
4. **Recommendations for RISEGAL**

Search for additional information: Yes/No

Experts advice needed: Yes/No

Experimental work needed: Yes/No

Research Project needed: Yes/No
