# Supplementary material for: Identification of Emerging Hazards in Mussels by the Galician Emerging Food Safety Risks Network (RISEGAL). A First Approach
Source: Foods. 2020 Nov 10;9(11):1641. doi: 10.3390/foods9111641 (PMC7697966; doi:10.3390/foods9111641)
Supplement: Supplementary file 1 [file foods-09-01641-s001.zip › Tables_figures_supplementary/Table S2_supplementary.docx]

| Table 2. List of *sources* included in the survey. |
| --- |
| **English sources:** Anses, Bill Marler - Twitter, Federal Office of Consumer Protection and Food Safety (BVL) - Official Control Germany, Cambro, Canadian Food Inspection Agency, Centers for disease control, DebHandHygiene - Twitter, Discovery Magazine, Earth eats, ECDC europa, EFSA, European Comission, Evira News, FDA, Food Insight, Food Poisoning Bulletin, Food Safe Guru, Food Safety Magazine, Food Safety News, Food Safety Tech, Food Standards Agency, FoodSafety, FSAI, Hazards Magazine, Health Canada, ICT Magazine, Institute of Food Technologists (IFT), lexblog.com, LiveScience, Medscape, MPI - New Zeland, Nafdac, National Geographic UK, New Food Magazine, PhysOrg, QA Magazine, Readers Digest, Science and Food UCLA, Science Daily, Science Magazine, The Ecologist, The Scientist, WebMD, World Fishing. |
| **Spanish sources:** Aetox.es, Agencia Catalana de Seguridad Alimentaria, Agencia española de Consumo, Seguridad alimentaria y Nutrición (AECOSAN), alimentatec.com, appccspain, argentina.gob.ar, Betelgeux, Boletín Epidemilógico de Castilla y León, Boletín Epidemilógico de Colombia, Boletín Epidemiloógico de Chile , Boletín Epidemiloógico de Galicia, Boletín Epidemiloógico de México, Boletín Epidemiológico de Perú, Boletín Epidemiológico de Semanal en Red - Instituto de Salud Carlos III, cienciadigital.es, CNTA, El Confidencial, EL País, Elika, Eroski Consumer, Europa Press, FIAB, invima.gov.co, Ministerio de Agricultura, Pesca y Alimentación, Revista Alimentaria, revista.nutricion.org, Scientia, scientificamerican.com, Servicio de Información y Noticias Científicas (SINC) , TecniFood techpress, toxicologia.org.ar, usa.gov (spanish). |
