# Supplementary material for: Identification of Emerging Hazards in Mussels by the Galician Emerging Food Safety Risks Network (RISEGAL). A First Approach
Source: Foods. 2020 Nov 10;9(11):1641. doi: 10.3390/foods9111641 (PMC7697966; doi:10.3390/foods9111641)
Supplement: Supplementary file 1 [file foods-09-01641-s001.zip › Tables_figures_supplementary/Table S3_supplementary.docx]

| Table 3. List of *searching groups* used by FoodRiskScan |
| --- |
| **Non-structured data bases (i.e. HTML type)**  Searching group 1: “agents”, “generic words” and “food products” |
| **Structured data bases (RSS and twitter)**  Searching group 2: “agents” and “food products”  Searching group 3: “generic words” and “food products” |
