# Supplementary material for: Identification of Emerging Hazards in Mussels by the Galician Emerging Food Safety Risks Network (RISEGAL). A First Approach
Source: Foods. 2020 Nov 10;9(11):1641. doi: 10.3390/foods9111641 (PMC7697966; doi:10.3390/foods9111641)
Supplement: Supplementary file 1 [file foods-09-01641-s001.zip › Tables_figures_supplementary/Table S4_supplementary.docx]

| Table 4. Keywords used in the scientific literature review |
| --- |
| **General search**. Period 2014-2018 |
| *Key words: emerg** and *food safety** and (seafood or shellfish or bivalve* or molluscs* or mussel* or clam or oyster*) |
| **Specific search**. Period 2016-2019 |
| *Key words:*  **virus** “Emerg*” and “vir*” and “food*” and (seafood or shellfish or bivalve* or mollus* or mussel* or mollus* or clam* or oyster*)  **Parasites:** (“parasit*” and "emerg*" and “food*” and (seafood or shellfish or bivalve* or mussel* or mollus* or clam* or oyster*)).  **chemical** (“contaminant*” or “pollutan*” or “chemical*” or “pharmaceutic*” or elemen*) and "emerg*" and “food*” and (seafood or shellfish or bivalve* or mussel* or mollus* or clam* or oyster*)).  **Bacteria:** “vibrio*” and “emerg*” and “food*” and (seafood or shellfish or bivalve* or mollus* or mussel* or mollus* or clam* or oyster*).“clostridi*” and “emerg*” and “food*” and (seafood or shellfish or bivalve* or mollus* or mussel* or mollus* or clam* or oyster*).“escherichia*” and “emerg*” and “food*” and (seafood or shellfish or bivalve* or mollus* or mussel* or mollus* or clam* or oyster*).“salmonel*” and “emerg*” and “food*” and (seafood or shellfish or bivalve* or mollus* or mussel* or mollus* or clam* or oyster*).“arcobacte*” and “emerg*” and “food*” and (seafood or shellfish or bivalve* or mollus* or mussel* or mollus* or clam* or oyster*).“lactococ*” and “emerg*” and “food*” and (seafood or shellfish or bivalve* or mollus* or mussel* or mollus* or clam* or oyster*).“listeri*” and “emerg*” and “food*” and (seafood or shellfish or bivalve* or mollus* or mussel* or mollus* or clam* or oyster*).  **biotoxins**: ("poison*" or toxin*) and emerg* and (food*) and (seafood or shellfish or bivalve* or mussel* or mollus* or clam* or oyster*).  **antimicrobial resistance:** (multidrug* or drug* or antibiotic* or antimicrob*) and "resistan*" and emerg* and food* and (seafood or shellfish or bivalve* or mussel* or mollus* or clam* or oyster*) |
