# Supplementary material for: Identification of Emerging Hazards in Mussels by the Galician Emerging Food Safety Risks Network (RISEGAL). A First Approach
Source: Foods. 2020 Nov 10;9(11):1641. doi: 10.3390/foods9111641 (PMC7697966; doi:10.3390/foods9111641)
Supplement: Supplementary file 1 [file foods-09-01641-s001.zip › Tables_figures_supplementary/Table S5_supplementary.docx]

| **Table 5. MRM conditions (DP: Declustering Potential; CE: Collision Energy)** | | | | |
| --- | --- | --- | --- | --- |
| **Antibiotic (target)** | **Q1 (m/z)** | **Q3 (m/z)** | **DP (V)** | **CE (V)** |
| **Amoxicillin** | 366.1 | 349.1 | 52 | 13 |
|  |  | 114.1 | 52 | 34 |
| **Ampicillin** | 348 | 207 | -45 | -18 |
|  |  | 173 | -45 | -28 |
| **Penicillin G** | 333.0 | 192.4 | -36 | -17 |
|  |  | 171.0 | -36 | -20 |
| **Cephalexin** | 348.0 | 174.1 | 90 | 21 |
|  |  | 158.0 | 90 | 15 |
| **Cefquinone sulphate** | 529.3 | 396.2 | 95 | 20 |
|  |  | 324.3 | 95 | 20 |
| **Chloramphenicol** | 321.1 | 194.0 | -65 | -17 |
|  |  | 152.0 | -61 | -23 |
| **Chlortetracycline** | 479.3 | 444.0 | 82 | 30 |
|  |  | 154.1 | 84 | 40 |
| **Ciprofloxacin** | 332.2 | 314.2 | 76 | 31 |
|  |  | 231.2 | 77 | 51 |
| **Cloxacillin** | 434.1 | 292.9 | -42 | -18 |
|  |  | 257.0 | -42 | -27 |
| **Dicloxacillin** | 469.9 | 160.0 | 106 | 19 |
|  |  | 113.9 | 106 | 47 |
| **Doxycycline** | 445.2 | 428.3 | 60 | 29 |
|  |  | 339.2 | 60 | 41 |
| **Enrofloxacin** | 360.3 | 342.2 | 85 | 31 |
|  |  | 245.1 | 85 | 39 |
| **Erythromycin** | 734.6 | 158.2 | -53 | -25 |
|  |  | 119.0 | -53 | -43 |
| **Florfenicol** | 356.1 | 185.0 | -53 | -25 |
|  |  | 119.0 | -53 | -43 |
| **Florfenicol Amine** | 248.1 | 230.2 | 50 | 20 |
|  |  | 130.1 | 50 | 35 |
| **Flumequine** | 262.1 | 202.1 | 67 | 47 |
|  |  | 244.1 | 67 | 29 |
| **Lincomycin** | 407.4 | 126.1 | 12 | 29 |
|  |  | 82.1 | 72 | 123 |
| **Lomefloxacin** | 352.2 | 265.2 | 67 | 36 |
|  |  | 308.3 | 67 | 25 |
| **Marbofloxacin** | 363.0 | 320.1 | 81 | 27 |
|  |  | 121.9 | 81 | 79 |
| **Nalidixic acid** | 232.9 | 215.0 | 56 | 21 |
|  |  | 186.9 | 56 | 21 |
| **Oxolinic acid** | 261.1 | 244.0 | 48 | 27 |
|  |  | 216.1 | 48 | 41 |
| **Oxytetracycline** | 461.2 | 426.1 | 55 | 39 |
|  |  | 201.1 | 55 | 54 |
| **Sulfadiazine** | 251.1 | 156.0 | 57 | 23 |
|  |  | 92.1 | 59 | 37 |
| **Sulfadimethoxine** | 311.1 | 156.2 | 74 | 26 |
|  |  | 92.1 | 73 | 50 |
| **Sulfadimine** | 278.9 | 155.9 | 56 | 23 |
|  |  | 107.9 | 56 | 25 |
| **Sulfathiazole** | 256.1 | 156.1 | 70 | 21 |
|  |  | 92.1 | 68 | 42 |
| **Tetracycline** | 445.2 | 410.1 | 62 | 27 |
|  |  | 154.1 | 62 | 40 |
| **Trimethoprim** | 291.1 | 230.1 | 95 | 33 |
|  |  | 123.1 | 95 | 34 |
| **Tylosin** | 916.7 | 174.3 | 59 | 54 |
|  |  | 88.3 | 59 | 117 |
