# Supplementary material for: Identification of Emerging Hazards in Mussels by the Galician Emerging Food Safety Risks Network (RISEGAL). A First Approach
Source: Foods. 2020 Nov 10;9(11):1641. doi: 10.3390/foods9111641 (PMC7697966; doi:10.3390/foods9111641)
Supplement: Supplementary file 1 [file foods-09-01641-s001.zip › Tables_figures_supplementary/Table S6_supplementary.docx]

Table 6: HILIC chromatographic conditions for TTX analysis.

| **Time (min)** | **A (%)** | **B (%)** | **Flow Rate (mL/min)** |
| --- | --- | --- | --- |
| 0.0 | 2.0 | 98.0 | 0.4 |
| 5.0 | 2.0 | 98.0 | 0.4 |
| 7.5 | 50.0 | 50.0 | 0.4 |
| 9.0 | 50.0 | 50.0 | 0.5 |
| 9.5 | 5.0 | 95.0 | 0.5 |
| 9.8 | 2.0 | 98.0 | 0.8 |
| 10.6 | 2.0 | 98.0 | 0.8 |
