# Supplementary material for: Identification of Emerging Hazards in Mussels by the Galician Emerging Food Safety Risks Network (RISEGAL). A First Approach
Source: Foods. 2020 Nov 10;9(11):1641. doi: 10.3390/foods9111641 (PMC7697966; doi:10.3390/foods9111641)
Supplement: Supplementary file 1 [file foods-09-01641-s001.zip › Tables_figures_supplementary/Table S7_supplementary.docx]

Table 7: MS/MS conditions (MRM mode) for the analysis of TTX and TTX analogues.

| **Compound** | **Precursor Ion** | **Product Ion** | **Collision Energy (V)** |
| --- | --- | --- | --- |
| TTX/4-epi-TTX | 320.1 | 302.1 | 28 |
| TTX/4-epi-TTX | 320.1 | 162.1 | 44 |
| 11-deoxy-TTX/5-deoxy-TTX | 304.1 | 286.1 | 28 |
| 11-deoxy-TTX/5-deoxy-TTX | 304.1 | 162.1 | 44 |
| 4,9-Anhydro TTX | 302.1 | 284.1 | 28 |
| 4,9-Anhydro TTX | 302.1 | 162.1 | 44 |
| 6,11-dideoxy-TTX | 290.1 | 272.1 | 28 |
| 6,11-dideoxy-TTX | 290.1 | 162.1 | 44 |
| 5,6,11-trideoxy-TTX | 272.1 | 254.1 | 28 |
| 5,6,11-trideoxy-TTX | 272.1 | 162.1 | 44 |
