# Supplementary material for: Identification of Emerging Hazards in Mussels by the Galician Emerging Food Safety Risks Network (RISEGAL). A First Approach
Source: Foods. 2020 Nov 10;9(11):1641. doi: 10.3390/foods9111641 (PMC7697966; doi:10.3390/foods9111641)
Supplement: Supplementary file 1 [file foods-09-01641-s001.zip › Tables_figures_supplementary/Table S8_supplementary.docx]

Table 8: iFunnel and Jetstream ion source conditions for TTX analysis.

| **Source Parameters** | | | |
| --- | --- | --- | --- |
| Gas temp (*◦*C) | 150 | Polarity | Positive |
| Gas flow (L/min) | 12 | Fragmentor (V) | 380 |
| Nebulizer (psi) | 45 | Cell accelerator (V) | 5 |
| Sheath gas heater (*◦*C) | 400 | Dwell (ms) | 20 |
| Sheath gas flow (L/min) | 12 | Delta EMV (V) | 400 |
| Capillary (V) | 4000 |  |  |
| V charging (V) | 300 |  |  |
| Ion Funnel Parameters (V) | | | |
| Pos high pressure RF | 150 | Neg high pressure RF | 90 |
| Pos low pressure RF | 60 | Neg low pressure RF | 60 |
