# Supplementary material for: Identification of Emerging Hazards in Mussels by the Galician Emerging Food Safety Risks Network (RISEGAL). A First Approach
Source: Foods. 2020 Nov 10;9(11):1641. doi: 10.3390/foods9111641 (PMC7697966; doi:10.3390/foods9111641)
Supplement: Supplementary file 1 [file foods-09-01641-s001.zip › Tables_figures_supplementary/Table S9_supplementary.docx]

| Table 9. Metrics rendered by the online inquiry | | | | |
| --- | --- | --- | --- | --- |
| Responses | Total visits | Unique visits | Ended inquiries (%)  of responses ended | Estimated  time to finish (min) |
| 50 | 214 | 122 | 41% | 2:48 |
